# Assessment of Tolerance to Lanthanum and Cerium in *Helianthus Annuus* Plant: Effect on Growth, Mineral Nutrition, and Secondary Metabolism

**DOI:** 10.3390/plants11070988

**Published:** 2022-04-05

**Authors:** Nesrine Dridi, Renata Ferreira, Houda Bouslimi, Pedro Brito, Susete Martins-Dias, Isabel Caçador, Noomene Sleimi

**Affiliations:** 1 LR. RME-Resources, Materials and Ecosystems, Faculty of Sciences of Bizerte, University of Carthage, Bizerte 7021, Tunisia; nesrinedridi442@yahoo.fr (N.D.); houda0702@hotmail.com (H.B.); 2 CERENA, Centro de Recursos Naturais e Ambiente, Instituto Superior Técnico, Universidade de Lisboa, 1049-001 Lisboa, Portugal; renata.ferreira@tecnico.ulisboa.pt; 3 IPMA, Division of Oceanography and Marine Environment, Instituto Português do Mar e da Atmosfera, 1495-165 Algés, Portugal; pbrito@ipma.pt; 4 CIIMAR—Interdisciplinary Centre of Marine and Environmental Research, 4450-208 Matosinhos, Portugal; 5 CERENA, Centro de Recursos Naturais e Ambiente, Departamento de Bioengenharia, Instituto Superior Técnico, Universidade de Lisboa, 1049-001 Lisboa, Portugal; susetedias@tecnico.ulisboa.pt; 6 MARE-FCUL, Centro de Ciências do Mar e do Ambiente, Faculdade de Ciências da Universidade de Lisboa, 1749-016 Lisboa, Portugal; micacador@fc.ul.pt

**Keywords:** REE uptake, lanthanum, cerium, mineral absorption, secondary metabolism, tolerance index

## Abstract

Rare earth elements (REEs) present a group of nonessential metals for the growth and development of plants. At high concentrations, they can induce internal stress and disturb the physiological and biochemical mechanisms in plants. The potential uptake of lanthanum (La) and cerium (Ce) by the horticultural plant *Helianthus annuus* and the effect of these elements on its growth, its absorption of macroelements, and the contents of phenolic compounds and flavonoids were assessed. The plants were exposed to 0, 1, 2.5, 5, and 10 µM of La and Ce for 14 days. The results showed a remarkable accumulation of the two REEs, especially in the roots, which was found to be positively correlated with the total phenolic compound and flavonoid content in the plant shoots and roots. The plant’s growth parameter patterns (such as dry weight and water content); the levels of potassium, calcium, and magnesium; and the tolerance index varied with the concentrations of the two studied elements. According to the tolerance index values, *H. annuus* had more affinity to La than to Ce. Although these metals were accumulated in *H. annuus* tissues, this *Asteraceae* plant cannot be considered as a hyperaccumulator species of these two REEs, since the obtained REE content in the plant’s upper parts was less than 1000 mg·Kg^−1^ DW.

## 1. Introduction

Rare earth elements (REEs), naturally abundant in the Earth’s crust and rocks [1], are known as a group of nonessential and nonbeneficial chemical elements in biological and physiological processes, including the development cycle of plants. Therefore, their excess anthropogenic use in different fields could engender severe environmental pollution and damage the balance of ecosystems [2,3]. Nevertheless, it has been established that the application of REEs as agricultural fertilizers promoted crop growth and increased yields [4,5]. Moreover, many research works have focused on the behavior of plants stressed by REEs, and it has been found that some of these metals could promote plant growth and protect plants against abiotic stress, such as acid rain, ultraviolet-B radiations [6], mineral uptake disturbance [7], and salinity [8]. Nonetheless, studies that focus on the interactions of REEs in soils and their effects on plant species and the environment are still limited [9].

An REE’s influence may be related to many factors, such as the cultivated species; the specific applied REE and its concentration; the developmental stage; the period of treatment; and the growth conditions (photoperiod, temperature, humidity, and watering frequency), which could positively or negatively affect the physiological metabolism of the plant [10,11]. In addition, the availability of REEs in soils could increase with a decrease in the pH and the redox potential [12].

Indeed, REE uptake by plants can engender oxidative stress, caused by the excessive production of reactive oxygen species (ROS), and result in severe damage to plants’ biological functions, leading to programmed cell death [10,13,14]. The stimulation of secondary metabolism to activate, e.g., phenolic compound and flavonoid production, is one of a plant’s cellular system strategies for defense against the metallic stress caused by REEs [14,15].

Lanthanum (La) and cerium (Ce), the most abundant members of the trivalent metallic element group (REEs) [1,16], are considered light REEs [17] that have a stable oxidation state (+3) and similar ionic radii to calcium and magnesium. This property can result in an inhibition of Ca^2+^ and Mg^2+^ uptake in plants, through their replacement by the REE in several physiological functions, and thus, in an imbalanced nutritional status, can limit their growth [10,16,18,19].

The ability of numerous plant species to accumulate more REEs in the underground parts than in the upper parts is well-known. This is due to the controlled redistribution of nutrient ions and other elements, such as REEs, through the apoplastic barriers in the roots [20]. These barriers present an obstacle to prevent these elements, when extremely available, from reaching the xylem; therefore, their translocation to the other plant parts is restrained [20]. The study of Brioschi et al. [21] also revealed that REEs might be retained by the Casparian strip of the roots, resulting in a very low translocation of these metals to the aerial parts.

An *Asteraceae* plant species, *Helianthus annuus,* was chosen for this study due to its high capacity to tolerate and hyperaccumulate a variety of heavy metals [22,23], and also because it has been identified as an effective species in the phytoremediation of polluted soils [24].

Aiming to better understand the behavior of this common sunflower in the presence of two specific REEs, the main objectives of this work were to study the accumulation potential of La and Ce in the shoots and roots of this species and to evaluate the phytotoxicity and tolerance ability to these elements. In this context, the effect of the accumulation of La and Ce in the plant on the growth of different plant parts, the mineral nutrition, and the biosynthesis of the secondary metabolite was assessed.

## 2. Material and Methods

### 2.1. Plant Culture and Exposure Experiments

The seeds used in the present work were collected from a cultivated field of sunflowers (*H. annuus*) situated in Béja in the North of Tunisia (36°49′90″ N, 9°13′77″ E). Selected *H. annuus* seeds were sterilized in hypochlorite calcium solution (5%) for 20 min and then rinsed three times and soaked for 2 h in distilled water. Afterwards, seeds were placed in petri dishes coated internally with soaked filter paper for five days at 25 °C in a germination oven. The obtained seedlings were transferred into a hydroponic system containing modified ¼ Hoagland nutrient solution [25] for five days before starting the treatment (to ensure their acclimatization) in a controlled-environment chamber (Fitoclima S600-Aralab, Portugal) under controlled conditions (photoperiod 12/12 h, mean temperature 25 ± 5 °C, and relative humidity between 60% and 80%).

After the adaptation period, two treatment experiments were conducted separately in a hydroponic system: one with La and the other with Ce. At the beginning of the treatment, grown seedlings were selected and divided into 9 groups of 24 seedlings, then placed in separate dark-walled 50 mL polypropylene tubes containing the nutritive solution supplemented with La or with Ce at 0, 1, 2.5, 5, and 10 µM (prepared from lanthanum and cerium certified reference standard solutions, 1000 mg·L^−1^ of La or Ce, high purity, HNO_3_ 2% (*v*/*v*), Sigma Aldrich, St Louis, CA, USA). The exposure trials were performed for 14 days. All treatment solutions were renewed daily to ensure that the initial concentrations were maintained throughout the exposure period. At the end of the experiments, treated plants were collected and split into two parts: shoots and roots. The shoots were rinsed with distilled water, while the roots were rinsed with CaCl_2_ 5% [26,27,28] to remove trace elements adsorbed on the root surface and washed twice with distilled water.

### 2.2. Dry Matter, Water Status, and Heavy Metal Tolerance Index Determination

Fresh weight (FW) of shoots and roots was determined immediately after the plants’ collection. After 7 days of plant tissue oven drying at 70 ± 2 °C, the dry weight (DW) was determined, which allowed the calculation of the water content (WC) [29], the shoot/root dry biomass ratio (S/R), and the REE tolerance index percentage (TI %) [30], following Equations (1)–(3):

(1) $$\mathrm{WC}\left(\mathrm{mL}/g\;\mathrm{DW}\right)=\frac{\left(\mathrm{Fresh}\;\mathrm{weight}-\mathrm{Dry}\;\mathrm{weight}\right)}{\mathrm{Fresh}\;\mathrm{weight}}$$

S/R = DW _Shoots_/DW _Roots_ (2)

(3) $$\mathrm{TI}\;\left(\%\right)=\frac{{\mathrm{DW}}_{\mathrm{Treated}\;\mathrm{plants}}}{{\mathrm{DW}}_{\mathrm{Untreated}\;\mathrm{plants}}}\times 100$$

### 2.3. REEs and Minerals Analysis

For the determination of K, Ca, Mg, La, and Ce concentrations in the plant tissues, dry plant material was reduced to a fine powder in an agate mortar. Thereafter, 30 mg of each sample was digested in the oven with a volume of 3 mL of a concentrated acid mixture composed of nitric acid, sulfuric acid, and perchloric acid (1.5:1:0.5 *v*/*v*/*v*) in Teflon vessels at 110 °C for two hours. The obtained digested products were filtered with Whatman filter (N°1) and diluted with nitric acid (0.5%) to reach 50 mL of total sample volume [29,31].

The K, Ca, and Mg concentrations were determined by atomic absorption spectrometry (AAS; Perkin Elmer PinAAcle 900T, Waltham, MA, USA. Copper was used as the AAS internal standard, and calibration standards from 0.5 to 5 mg·L^−1^ were prepared from the Ca, Mg, and K stock solutions (to be periodically run as unknowns to check recovery).

The quantification of La and Ce concentrations in *H. annuus* roots and shoots was determined by inductively coupled plasma mass spectrometry (ICP-MS; Perkin-Elmer NexION2000C), as described by Brito et al. [32].

The translocation factor (TF) of the metals was calculated following the equation [33]:

TF = REE concentration _Shoot_/REE concentration _Root_ (4)

### 2.4. Determination of Phenolic Compounds and Flavonoids

Approximately 30 mg of the dry sample was mixed in 10 mL of methanol 80% and settled overnight in the dark. The extract was centrifuged at 2500 rpm/min for 30 min and the supernatant was filtered. Total phenol content was assessed following the procedure of Velioglu et al. [34], optimized by Bouslimi et al. [35]. The determination of flavonoid content was accomplished as previously described by Quettier et al. [36].

### 2.5. Statistical Analysis

Obtained results were tested using one-way ANOVA and Tukey honest significant difference test (HSD). All data were presented as means ± standard deviation (SD) with n = 24, and results were considered significant at *p* < 0.01 and *p* < 0.05. Associations between the studied parameters were determined by simple correlation analysis (Pearson correlation) by Microsoft Excel.

## 3. Results and Discussion

### 3.1. Lanthanum and Cerium Influence on H. Annuus

#### 3.1.1. Plant Morphology and Growth

The observation of the morphology and the monitoring of the growth of *H. annuus* during and at the end of the treatment (14th day) with La and Ce revealed that the studied plant showed no reduction or enhancement in height (for all the applied REE concentrations when comparing to the untreated plants). Treated plants developed the same number of leaves as the control plants. Moreover, no visible signs of toxicity or foliar chlorosis and necrosis, or deficiency in essential elements, were noticed (see Figure S1 in Supplementary Materials).

**Figure 1:**
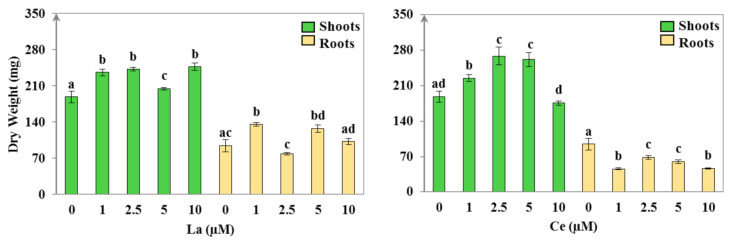
Dry weight of shoots and roots of *Helianthus annuus* after 14 days of treatment with different concentrations of lanthanum (La) and cerium (Ce). Values are mean ± SD (n = 24). Within each treatment group and plant organ, values with different lower-case letters are significantly different (*p* < 0.05).

After 14 days of La or Ce exposure, the dry biomass of the *H. annuus* plants showed significant variations when treated with different concentrations of REE (Figure 1). The dry weight increased significantly in the shoots (*p* < 0.01) as the La concentration increased. Moreover, the association between the accumulated La content and DW content in the shoots showed a significant positive correlation (*p* < 0.05; Table S1).

In the roots, the DW increased only in the plants treated with 1 µM and 5 µM of La, when compared to the control. Even though the plants treated with 10 µM of La also showed a slight increase in their root biomass, it was not significantly different (*p* > 0.05) when compared to the control. Moreover, an insignificant decrease in the roots of the plants that were exposed to 2.5 µM of La was noticed. Contrarily, Ce-treated plants showed a significant dry weight increase (*p* < 0.01) in the shoots for the 1, 2.5, and 5 µM treatments in comparison to the control plants. The shoots of the plants treated with 10 µM of Ce were not significantly affected (*p* > 0.05). However, the roots’ DW was significantly reduced (*p* < 0.05) for all applied Ce concentrations when compared to the control (Figure 1); in addition, the Ce content in roots was found to be negatively correlated with the DW (*p* < 0.05; Table S2). The obtained results are in line with those of Oliveira et al. [5] and Chen et al. [10], who demonstrated that La and Ce could promote the growth of soybean and ginkgo plants, respectively. Moreover, the same pattern of results was reported for the *Brassica chinensis* plant growing in the presence of La and Ce [37].

The plant growth enhancement is related to the increase in the mitotic index, which can be stimulated by the presence of low concentrations of La in the growth medium [5]. A low La concentration might promote plant cell division and proliferation, since it can induce a high incidence of binucleate cells without trigging a chromosomal abnormality emergency that blocks cell division, such as in the C-metaphase [5,38]. However, all plant toxicity symptoms (caused by trace elements) were induced due to the induction of chromosomal aberrations that ultimately lead to a reduction in the mitotic index [39]. This fact may explain the decrease in the root dry biomass in Ce plants. Moreover, the root growth decline might be due to the increase in the REE uptake, which induces metabolic function changes [40]. In fact, Vachirapatama et al. [41] reported that metals such as vanadium can inhibit the plasma membrane hydrogen H^+^-ATPase, which is involved in nutrient element uptake by plant cells.

#### 3.1.2. Tolerance Index

The analysis of the La tolerance index percentage (Table 1) showed that *Helianthus* experienced a notable amelioration in shoots (up to 99%) and the entire plant (EP) (up to 72%) in all the La treatments, and in the roots (up to 64%) for the plants exposed to 1, 5, and 10 µM of La, whereas in the roots of plants exposed to 2.5 µM of La, a 25% reduction occurred.

The tolerance index was also higher in the shoots (up to 143%) of *Helianthus* treated with 1, 2.5, and 5 µM of Ce, whilst the shoots of the plants exposed to 10 µM of Ce decreased by 22.3%. Moreover, for all the applied Ce concentrations, a reduction was observed in the roots (up to 76%). Regarding the results for the EP, an increase was noticed in the plants treated with 2.5 and 5 µM of Ce (up to 44%), and a 50% decrease for 10 µM of Ce.

Overall, the TI of the entire plant indicated that *H. annuus* plants have a higher tolerance to La than to Ce (Table 1). Specifically, the plants’ shoot and root TI values showed that *H. annuus* tolerated the presence of La in the medium, since both organs presented a TI greater than 100% (except the roots of the plants treated with 2.5 µM of La). Contrarily, the TI of plants exposed to Ce was lower than 100% in the roots for all the tested concentrations, which indicated that the plant was under stress and its tolerance to this specific metal is lower [29]. The tolerance of plants to metallic stress can be explained by their ability to activate mechanisms such as the detoxification, sequestration, or translocation of the metallic elements, or by their ability to concentrate excess metals in their bio-inactive cells to minimize, or even avoid, tissue toxicity [3,29].

#### 3.1.3. Water Status

The REE treatments had a slight effect on the WC of the *H. annuus* organs (Table 1). This effect was more noticeable in the roots, especially in plants treated with Ce (for all the Ce concentrations). The WC in *Helianthus* shoots significantly decreased (*p* < 0.01) in plants treated with 10 µM of Ce, whereas a significant increase (*p* < 0.01) in the roots of all Ce-treated plants was detected when compared to the control. Compared to the untreated plants, the water status in the two studied organs of La-treated plants showed no significant variation (*p* > 0.05) in the shoots, but an increase in the roots of the plants exposed to 2.5 (*p* < 0.01) and 10 µM (*p* < 0.05) of La was observed (Table 1). Our results might be explained by a probable water balance disturbance due to the disruption of the membrane permeability (which led to an increase in the root WC). Kohli et al. [42] reported similar results in *H. annuus* growing in the presence of Pb (a decline in TI (%) and DW was also observed). However, the reduction in water supply in the plants might be related to the downregulation of aquaporin genes under REEs, whereas decreased aquaporin and reduced water permeability can be a defense mechanism adopted by plants in order to allow a greater conservation of cellular water and improve the tolerance to abiotic stresses [43].

### 3.2. REE and Mineral Uptake by H. Annuus

#### 3.2.1. La and Ce Accumulation

**Figure 2:**
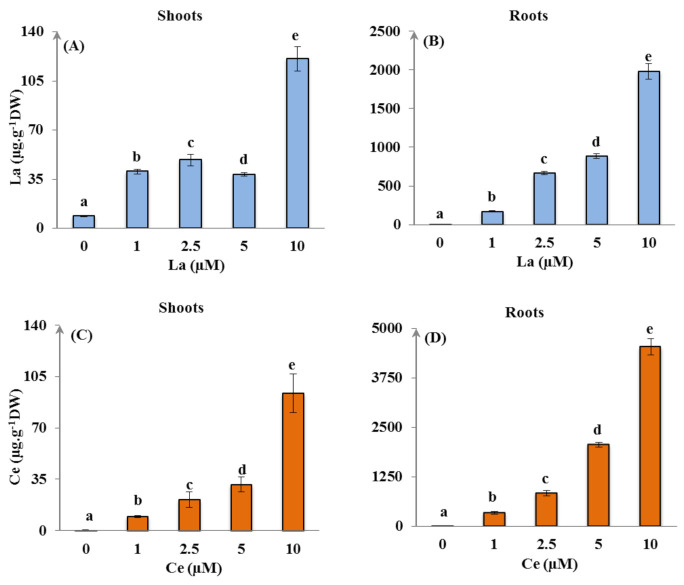
Lanthanum (**A**,**B**) and cerium (**C**,**D**) concentrations in *Helianthus annuus* shoots and roots after 14 days of metal exposure (values are mean ± SD, n = 24). Within the treatment group and plant organ, values with different letters are significantly different (*p* < 0.01).

The La and Ce concentrations accumulated in the different organs of the studied *Asteraceae* plant at the different tested concentrations are presented in Figure 2. The results clearly showed that the concentrations of La and Ce in the plant roots were higher than in the shoots. It was noticed that the La and Ce accumulated in the plant organs was correlated to the increase of the REE-concentrations in the hydroponic medium (*p* < 0.01).

The concentrations of La and Ce in the shoots also demonstrated a significant increase compared to the control (*p* < 0.05), with a very low translocation of these elements from the root to the shoot (the translocation factor ratio was less than 1; Table 2).

The results showed that La and Ce uptake increased in *H. annuus* shoots and roots with the increasing concentrations of REEs in the nutritive solutions in comparison to the control (Figure 2), with the roots being the preferential REE accumulation organ. The obtained results are in agreement with the trials of Rezaee et al. [44], whose results showed that the accumulated La and neodymium (Nd) were higher in the roots of *H. annuus* and *Brassica chinensis*. Milania et al. [13] also demonstrated that Ce accumulation is usually lower in shoots. Since a metal hyperaccumulator plant should highly tolerate and accumulate more than 1000 mg·kg^−1^ of a metal in its aerial part and should have a translocation factor greater than 0.2 [44], *H. annuus* could not be considered as a potential hyperaccumulator species of La and Ce under the tested conditions. In fact, generally, to reduce metal toxicity, plants can minimize the metal translocation from the root system to the aerial parts [44], and the elevated retention of toxic metals in roots presents an acquired tolerance strategy in plants in order to protect the photosynthetic apparatus.

#### 3.2.2. Mineral Uptake

**Figure 3:**
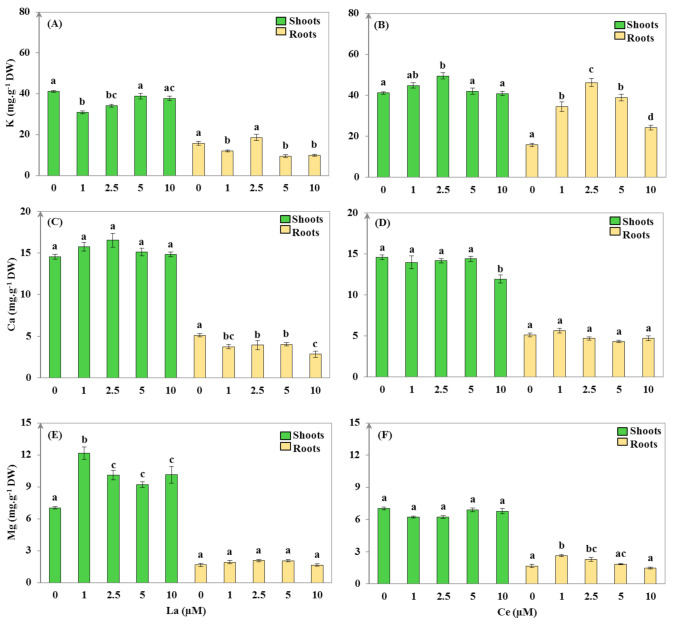
Macronutrient uptake ((**A**,**B**)—potassium (K); (**C**,**D**)—calcium (Ca); (**E**,**F**)—magnesium (Mg)) in shoots and roots of *Helianthus annuus* after 14 days of treatment with different concentrations of lanthanum (La) and cerium (Ce) (values are mean ± SD, n = 24). Within the treatment group and plant organ, values with different lower-case letters are significantly different (*p* < 0.05).

As can be seen in Figure 3, the presence of the different concentrations of REEs in the medium affected the mineral absorption by *H. annuus*. The absorption of K significantly decreased in the shoots of plants treated with 1 and 2.5 µM of La, whereas no differences were noticed for the 5 and 10 µM doses (*p* > 0.05) when compared to the control (Figure 3A). Moreover, a significant reduction in K was also found in the roots of the 1, 5, and 10 µM La concentration plants (Figure 3B). Contrarily, Ce caused a significant increase in K absorption only in the 2.5 µM shoots and a significant increase in the roots for all the Ce treatments, with the highest absorption detected for the 2.5 µM concentration (Figure 3A,B).

In comparison to the control, none of the La treatments showed any significant differences in the Ca concentration in the shoots (Figure 3C). However, the Ca contents in the roots significantly decreased (*p* < 0.05; Figure 3C). Contrarily to the La effect, Ce did not induce any significant variation in the Ca absorption in roots and shoots, except in the shoots of plants treated with 10 µM of Ce (18% reduction when compared to the untreated plants; Figure 3D). A significant negative correlation between La accumulated in roots and both K and Ca was observed (*p* < 0.05; Table S1).

The results showed a significant Mg increase (*p* < 0.05) in the shoots of *Helianthus* plants treated with La (the highest absorption was noticed in the 1 µM dose; Figure 3E). The magnesium absorption was not affected in the roots (Figure 3E). However, in the presence of Ce, plants behaved differently in terms of Mg uptake: no significant variations were noted in the shoots, whereas an enhancement (*p* < 0.05) was observed in the roots of plants treated with 1 and 2.5 µM of Ce when compared to the control (Figure 3F).

It is well-known that macronutrients, such as Mg, Ca, and K, can interfere with chloroplast functions, such as protein synthesis, the activation of the enzyme Mg^2+^ATPase, the stabilization of the membrane chloroplast structure, and the osmotic potential, and that REE accumulation may influence their uptake [45,46,47].

Several studies have shown that plants respond differently to Ce addition in terms of nutriment regulation in their tissues. Hu et al. [40] reported a decrease in Ca in the shoots and roots of wheat plants and a reduction in the K levels in roots, contrarily to maize plants, where Ce decreased Ca and enhanced Mg in shoots without affecting these element concentrations in the roots [40]. Ca and Mg absorption also increased in the shoots of mungbean [11]. Moreover, Ginkgo plants did not show a significant variation in Mg uptake in comparison to the untreated plants, but Ca significantly decreased with varied doses of Ce [10]. In the shoots of rice species, macroelement uptake was not influenced by the supplied Ce; however, there was a significant increase in the Mg content in roots of plants exposed to 50 µM of Ce, and a Ca content decline in the plants treated with 25 and 50 µM [11].

These results can be explained by the synergistic and antagonistic relationships between REEs and essential nutrients [9,11]. Because of their similar physicochemical characterization, REEs such as Ce and La might cause the blockage of Ca^2+^ ionic channels and inhibit the absorption of these ions through the Ca channels in plants [48,49,50,51]. Membrane stability and cell ionic interactions might be affected by these elements [11,52]. Moreover, La and Ce might bind to membrane proteins in the chloroplast, resulting in an alteration of protein forms, inducing a reduction in Mg^2+^ATPase activity [45].

Shyam et al. [53] and Chen et al. [10] have confirmed that some REEs could replace Mg^2+^ in chlorophyll formation by performing a catalytic function. Therefore, Ce and La can bind to chlorophyll to form Ce–chlorophyll or La–chlorophyll complexes instead of Mg–chlorophyll in chloroplast cells and thylakoïds. According to Zhang et al. [51], La can affect both protease physiological activity and plasma membrane redox system activity, as well as decreasing ion transportation; the authors reported an enhancement in K leakage in La-treated plants, probably due to membrane disruption. The results of Peralta-Videa et al. [45] and Hu et al. [46] revealed that low concentrations of La and Ce induced an enhancement in Ca, K, and Mg levels, although they were found to be reduced at high doses of REEs. Moreover, the high K uptake by the plants might also be explained by the fact that REEs can stimulate abscisic acid synthesis [54]. Previous studies showed that Mg can intervene in metal toxicity alleviation through the maintenance of the Fe status, the enhancement of the H+-ATPase activity and antioxidative capacity, the sequestration of metal ions in cellular compartments and the detoxification and the protection of the photosynthetic apparatus [55,56].

### 3.3. Secondary Metabolism Assessment

**Figure 4:**
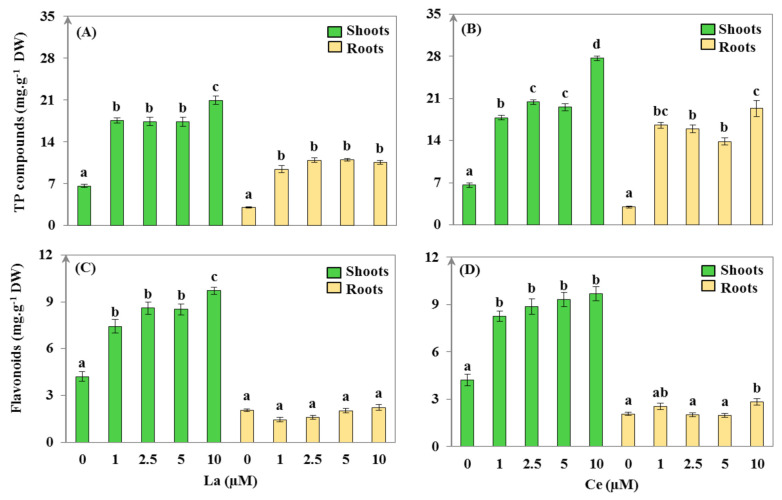
Total phenolic (TP) compound (**A**,**B**) and flavonoid (**C**,**D**) content in *Helianthus annuus* shoots and roots after 14 days of treatment with lanthanum (La) and cerium (Ce) (values are mean ± SD, n = 24). Within the treatment group and plant organ, values with different lower-case letters are significantly different (*p* < 0.01).

Biotic and abiotic stresses, including metallic stress, could enhance secondary metabolite production in plants to reduce the formation of ROS and avoid cell damage. In this work, and in comparison with the untreated plants, the levels of phenolic compounds in the shoots and roots under La and Ce stress significantly increased, by more than 50% (*p* < 0.01; Figure 4A,B). The La treatment also resulted in a significant increase in the flavonoid content in shoots, but no significant (*p* > 0.05) variation in roots was noticed (Figure 4C,D). Similarly, the flavonoid content significantly increased (*p* < 0.01) in the shoots of plants treated with different concentrations of Ce, but only a slightly significant enhancement was observed in the roots of the plants exposed to the 10 µM dose of Ce when compared to the untreated plants (Figure 4D). Indeed, correlation analysis showed that the uptake of La or Ce in shoots and roots was positively correlated with phenols and flavonoids (*p* < 0.05; Tables S1 and S2).

Our results are in agreement with the study of Babula et al. [57], where La-treated *Hypericum perforatum* plants showed a notable accumulation of phenolic acids in their shoots and roots. Michalak [58] stated that the application of high concentrations of metals induces the accumulation of phenols and flavonoids in plant tissues. Many researchers have explained the antioxidative capacity of phenols and flavonoids in chelating uptake metals and scavenging ROS by transferring electrons to free radicals [42,58,59,60]. Metals have the ability to deteriorate lipid hydroperoxide (LOOH) by the hemolytic cleavage of the (O-O) bond, resulting in lipid alkoxyl radicals and the establishment of free-radical chain oxidation. Phenolic compounds and flavonoids can trap lipid alkoxyl radicals to inhibit lipid peroxidation, depending on the number and position of the hydroxyl group in the molecules and its structure [58].

Chen et al. [10] and Bouslimi et al. [35] reported that REEs such as La, Ce, Nd, and yttrium were applied to stimulate the production of secondary metabolites (e.g., flavonoids, taxol, crocin, and catharanthine) in *Taxus, Tetrastigma hemsleyanum, Saussurea medusa,* and *Crocus sativus* plants. It was found that the presence of REEs in plant cells stimulate phenylalanineammonialyase (PAL) activity, which is the main enzyme responsible for phenolic compound and flavonoid biosynthesis. It was also revealed that the increase in PAL expression could explain the enhancement in the total phenol and flavonoid levels in plants [10,35].

Despite the high levels of phenols in the roots, which indicated that the plants were stressed in the presence of La and Ce, the flavonoid content did not change. This might indicate that flavonoids did not interfere in the plants’ defense against the metallic stress caused by these two REEs (except in the 10 µM Ce treatment) and that only phenols were involved. This may explain the strategy applied by this plant species to save energy by investing only in the production of phenols (hydroxycinnamic acids), while the genes intervening in the formation of flavonoids and anthocyanins stay deactivated [14].

## 4. Conclusions

In summary, the application of the studied REEs (La and Ce) on *Helianthus annuus* did not show severe negative effects on this species. In fact, the exposure to La promoted an increase in shoot and root dry biomass. However, in all the Ce treatments, the dry biomass of the shoots increased, while the root dry biomass was significantly reduced. Though the growth parameter patterns, such as the dry weight, water content, tolerance index, and shoot/root ratio, and the Ca, K, and Mg uptake varied with the concentrations of the two studied elements, no critical imbalance was noticed.

*H. annuus* preferentially accumulated La and Ce in the roots. Although this *Asteraceae* plant could highly accumulate these REEs in its belowground organs, it could not be considered as a hyperaccumulator species for La and Ce. Moreover, taking into account the tolerance index values, *H. annuus* showed more affinity to La than to Ce. The metal accumulation was found to be positively correlated with the total phenolic compound and flavonoid content in the plant shoots and roots. These findings proved that this species is susceptible to defending itself from induced stress and avoiding phytotoxicity through the activation of secondary metabolism. However, the assessment of some molecular analyses, such as phytochelatin synthesis and the expressed genes implicated in oxidative stress tolerance in response to REE toxicity, in addition to the determination of metal compartmentalization in the plant cells, would help to further the understanding of the tolerance and adaptation mechanisms adopted by this species to reduce La and Ce stress.

## Figures and Tables

**Table 1:** Water content (WC), shoot/root dry biomass ratio (S/R), entire plant (EP) dry weight (DW), and tolerance index (TI) of *Helianthus annuus* under exposure to different La and Ce concentrations. Values are mean ± SD (n = 24); (*) and (**) are significantly different at *p* < 0.05 and *p* < 0.01, respectively.

La (μM)	WC (mL·g^−1^ DW)		S/R	EP DW (mg)	TI (%)		
	Shoots	Roots			Shoots	Roots	EP
0	0.927 ± 0.002 0.925 ± 0.001 0.920 ± 0.001 0.924 ± 0.002 0.922 ± 0.002	0.943 ± 0.002	2.1 ± 0.2	283.2 ± 21.5			
1		0.939 ± 0.001	1.7 ± 0.0	371.4 * ± 8.8	180.6 ± 11.2	164.4 ± 5.7	172.2 ± 7.2
2.5		0.959 ** ± 0.000	3.1 ± 0.1	321.4 * ± 3.4	191.1 ± 6.1	75.8 ± 3.6	131.3 ± 2.8
5		0.939 ± 0.001	1.6 ± 0.1	332.2 * ± 7.4	127.4 ± 4.3	151.9 ± 11.1	140.1 ± 6.1
10		0.949 * ± 0.001	2.4 ± 0.1	349.4 * ± 11.9	199.1 ± 11.6	112.5 ± 9.3	154.2 ± 9.7
**Ce (μM)**	**WC (mL·g^−1^DW)**		**S/R**	**EP DW (mg)**	**TI (%)**		
	**Shoots**	**Roots**			**Shoots**	**Roots**	**EP**
0	0.927 ± 0.002 0.935 ± 0.003 0.937 ± 0.003 0.938 ± 0.001 0.907 ** ± 0.005	0.943 ± 0.002	2.1 ± 0.2	283.2 ± 21.5			
1		0.964 ** ± 0.002	4.9 ± 0.2	271.0 ± 7.5	162.0 ± 10.9	23.3 ± 2.9	90.0 ± 6.2
2.5		0.974 ** ± 0.002	4.0 ± 0.1	337.0 * ± 17.0	235.9 ± 29.3	58.9 ± 5.8	144.0 ± 13.9
5		0.973 ** ± 0.001	4.5 ± 0.2	321.3 * ± 14.1	242.9 ± 14.2	45.3 ± 6.2	131.2 ± 11.5
10		0.969 ** ± 0.002	3.8 ± 0.1	222.1* ± 4.2	77.7 ± 8.0	24.3 ± 1.7	50.0 ± 3.4

**Table 2:** Translocation factor ratio (TF) of La and Ce in *Helianthus annuus.* Values are mean ± SD (n = 24).

REE Concentration (µM)	TF	
	La	Ce
1	0.238 ± 0.019	0.028 ± 0.003
2.5	0.073 ± 0.009	0.026 ± 0.009
5	0.044 ± 0.002	0.015 ± 0.003
10	0.061 ± 0.009	0.021 ± 0.003

## Supplementary Materials

The following supporting information can be downloaded at: https://www.mdpi.com/article/10.3390/plants11070988/s1, Figure S1: An example of *Helianthus annuus* plant morphology after 14 days of REE exposure, Table S1: Correlations (Pearson correlation) between accumulated La in the different organs of *Helianthus annuus* and the studied parameters, Table S2: Correlation (Pearson correlation) between accumulated Ce in the different organs of *Helianthus annuus* and the studied parameters.
