# Supplementary material for: Assessment of Tolerance to Lanthanum and Cerium in Helianthus Annuus Plant: Effect on Growth, Mineral Nutrition, and Secondary Metabolism
Source: Plants (Basel). 2022 Apr 5;11(7):988. doi: 10.3390/plants11070988 (PMC9002919; doi:10.3390/plants11070988)
Supplement: Supplementary file 1 [file plants-11-00988-s001.zip › plants-1635031-supplementary.pdf]

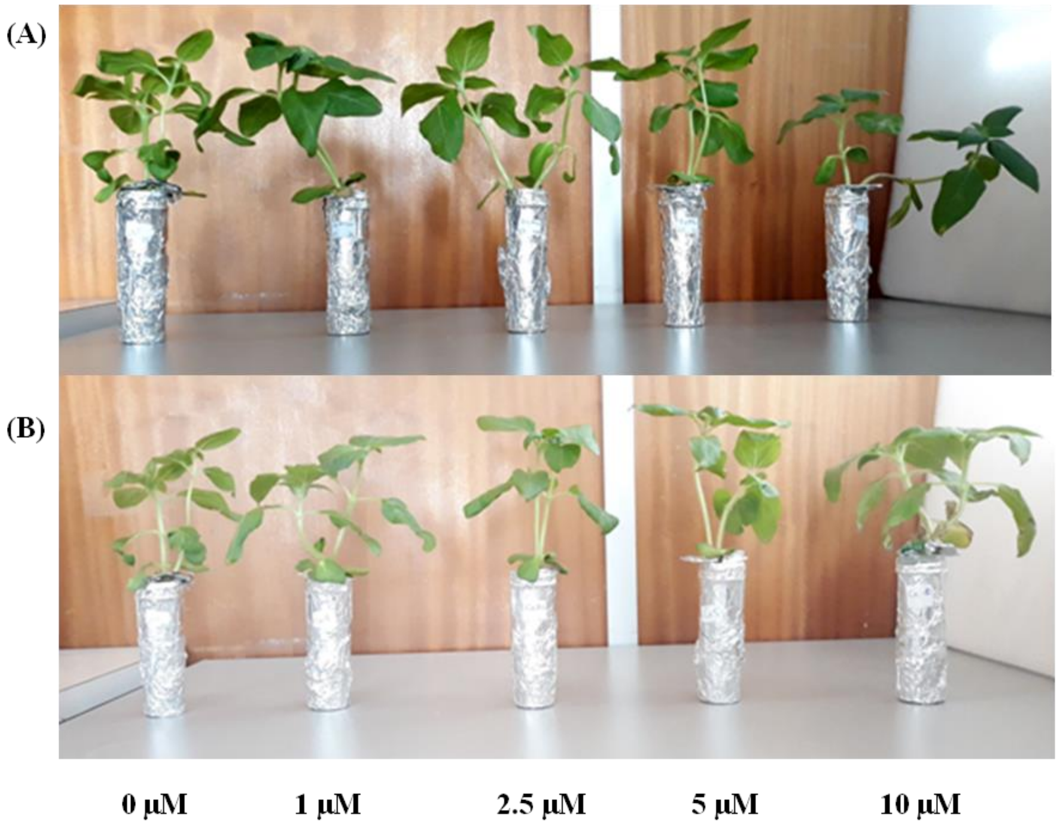

Figure S1. An example of *Helianthus annuus* plants morphology after 14 days of REEs exposure; (A): Lanthanum treated plants, (B): Cerium treated plants.

Table S1. Correlations (Pearson correlation) between accumulated La in the different organs of *Helianthus annuus* and the studied parameters.

**Shoots**	**La**	**TP**	**FLAV**	**DW**	**WC**	**K**	**Mg**	**Ca**
**La**	**1.000**							
**TP**	**0.778***	1.000						
**FLAV**	**0.792***	0.972*	1.000					
**DW**	**0.743***	0.831*	0.773*	1.000				
**WC**	**-0.271**	-0.635*	-0.522	-0.837*	1.000			
**K**	**-0.110**	-0.522*	-0.362	-0.704*	0.962**	1.000		
**Mg**	**0.304**	0.679*	0.499	0.722*	-0.873*	-0.932**	1.000	
**Ca**	**-0.055**	0.394	0.373	0.575	-0.851*	-0.772*	0.548	1.000

**Roots**	**La**	**TP**	**FLAV**	**DW**	**WC**	**K**	**Mg**	**Ca**
**La**	**1.000**							
**TP**	**0.586***	1.000						
**FLAV**	**0.575***	-0.215	1.000					
**DW**	**-0.105**	0.212	-0.169	1.000				
**WC**	**0.284**	0.262	-0.120	-0.862*	1.000			
**K**	**-0.564***	-0.429	-0.408	-0.734*	0.602*	1.000		
**Mg**	**-0.216**	0.599*	-0.608*	0.156	0.148	0.108	1.000	
**Ca**	**-0.828***	-0.781*	-0.076	-0.180	-0.209	0.578	0.017	1.000

TP: total phenolic compounds, FLAV: flavonoids content. Values with (*) and (**) present significant differences at $p < 0.05$ and $p < 0.01$, respectively.

Table S2. Correlation (Pearson correlation) between accumulated Ce in the different organs of *Helianthus annuus* and the studied parameters

**Shoots**	**Ce**	**TP**	**FLAV**	**DW**	**WC**	**K**	**Mg**	**Ca**
**Ce**	**1.000**							
**TP**	**0.838***	1.000						
**FLAV**	**0.636***	0.939	1.000					
**DW**	**-0.390**	0.077	0.380	1.000				
**WC**	**-0.800***	-0.405	-0.079	0.896	1.000			
**K**	**-0.344**	0.113	0.251	0.844	0.557	1.000		
**Mg**	**-0.937****	-0.770	-0.515	0.571	0.883	0.309	1.000	
**Ca**	**0.176**	-0.309	-0.407	-0.468	-0.369	-0.847	-0.024	1.000
**Roots**	**Ce**	**TP**	**FLAV**	**DW**	**WC**	**K**	**Mg**	**Ca**
**Ce**	**1.000**							
**TP**	**0.610***	1.000						
**FLAV**	**0.594***	0.557	1.000					
**DW**	**-0.564***	-0.926**	-0.670*	1.000				
**WC**	**0.473**	0.868*	0.084	-0.709	1.000			
**K**	**-0.093**	0.558*	-0.343	-0.385	0.829*	1.000		
**Mg**	**0.524**	-0.154	0.372	-0.039	-0.501*	-0.305	1.000	
**Ca**	**-0.612***	0.246	-0.119	-0.272	0.250	0.619*	0.544	1.000

TP: total phenolic compounds, FLAV: flavonoids content. Values with (*) and (**) present significant differences at $p < 0.05$ and $p < 0.01$, respectively.
